# ARID1A deficiency attenuates the response to EGFR-TKI treatment in lung adenocarcinoma

**DOI:** 10.3389/fphar.2025.1582005

**Published:** 2025-05-20

**Authors:** Fangfang Yang, Helei Hou, Guanqun Wang, Guangming Fu, Xingfa Huo, Xueqin Duan, Na Zhou, Xiaochun Zhang

**Affiliations:** ^1^ Precision Medicine Center of Oncology, The Affiliated Hospital of Qingdao University, Qingdao, China; ^2^ Qingdao Medical College, Qingdao University, Qingdao, China; ^3^ Department of Oncology, The Affiliated Hospital of Qingdao University, Qingdao, China; ^4^ Department of Pathology, The Affiliated Hospital of Qingdao University, Qingdao, China

**Keywords:** ARID1A, epidermal growth factor receptor, tyrosine kinase inhibitor, resistance, lung adenocarcinoma

## Abstract

**Background:**

Concurrent genetic alterations (e.g., TP53 comutations) significantly impair EGFR-TKI responsiveness and survival outcomes in EGFR-mutant lung adenocarcinoma (LUAD). AT-rich interactive domain 1A (ARID1A), which is a key subunit of SWI/SNF complexes, demonstrates critical regulatory functions as a tumour suppressor gene in cancer. The aim of this study is to determine the role of ARID1A deficiency in the therapeutic efficacy of EGFR-TKIs in LUAD.

**Methods:**

We identified the ARID1A mutation as a potential prognostic marker in EGFR-mutant LUAD by analysing data from cBioPortal. The expression of ARID1A was detected via immunohistochemical staining. A lentivirus was employed to construct the ARID1A knockdown model in PC9 cell. We further analyzed the biological roles of ARID1A knockdown through CCK8, flow cytometry analysis and transwell assay.

**Results:**

The ARID1A mutation was associated with poor OS in EGFR-mutant LUAD patients, and the prognostic influence was greater than that of concurrent EGFR mutations with TP53, KRAS, CDKN2A, PIK3CA, RB1 or PTEN. By analysing the clinical data of our centre, we revealed that patients with loss of ARID1A expression demonstrated poorer median progression-free survival (mPFS, 10.3 versus 30 months, *P* = 0.005) when they received EGFR-TKIs as the first-line treatment after postoperative progression (cohort A). A shorter median disease-free survival (mDFS, 29 versus NA months, *P* = 0.003) was also observed in the ARID1A low-expression cohort than in the ARID1A high-expression group in patients receiving postoperative adjuvant EGFR-TKI treatments (cohort B). We also found that ARID1A deficiency attenuated the efficacy of osimertinib by activating the EGFR/AKT/mTOR signalling axis in PC9 cell.

**Conclusion:**

ARID1A deficiency may be an independent prognostic factor and attenuates the response to EGFR-TKIs in patients with EGFR-mutant LUAD. In addition, loss of ARID1A expression confers resistance to EGFR-TKI by activating the EGFR/AKT/mTOR signalling axis.

## 1 Introduction

Lung cancer is the most prevalent malignant tumor and a leading cause of cancer-related deaths worldwide, with approximately 2.5 million new cases (12.4%) and 1.8 million deaths (18.7%) per year (Bray et al., 2024). Lung adenocarcinoma (LUAD) is the most common pathological type of non-small cell lung cancer (NSCLC) and accounts for more than 50% of cases (Succony et al., 2021). The identification of epidermal growth factor receptor (EGFR) mutations represents a breakthrough in the treatment paradigm for NSCLC. EGFR mutations have been shown to occur in more than 50% of LUAD patients in the Asian population (Kim et al., 2021; Grosse et al., 2019). The most common sensitizing EGFR mutations include in-frame exon 19 deletions and an exon 21 point mutation (L858R) (Levantini et al., 2022). Multiple phase III trials have validated that first-generation EGFR-TKIs (gefitinib, erlotinib, icotinib) and second-generation EGFR-TKIs (afatinib, dacomitinib) significantly improves objective response rate (ORR) and progression-free survival (PFS) compared to platinum-based chemotherapy in treatment-naïve NSCLC patients with EGFR mutations (Fukuoka et al., 2011; Rosell et al., 2012; Sequist et al., 2013). Osimertinib, a third-generation EGFR-TKI, achieves significant survival benefits compared to first-generation EGFR-TKIs, with increase PFS (18.9 versus10.2 months) and overall survival (OS) (38.6 versus 31.8 months) (Ramalingam et al., 2020; Soria et al., 2018). The clinical application of EGFR-TKIs significantly prolongs the survival of patients. However, acquired resistance limits the long-term clinical efficacy of EGFR-TKIs.

Several studies have shown that concurrent genetic alterations, such as TP53 mutations (which is the most frequently comutated gene), exert a negative effect on the response to EGFR-TKIs and the prognosis of EGFR-mutant LUAD (Kim et al., 2019). Nevertheless, specific concurrent genetic alterations affecting the efficacy of EGFR-TKIs are not clear. Thus, it is necessary to further investigate the mechanisms of EGFR-TKI resistance in order to optimize the treatment schedule. The switch/sucrose nonfermenting (SWI/SNF) complex, which represents a subfamily of ATP-dependent chromatin remodelling complexes, plays important roles in chromatin recombination, gene regulation and DNA damage repair as tumour suppressors (Malone and Roberts, 2024). The genes encoding SWI/SNF subunits are mutated in more than 20% of cancers (Centore et al., 2020), which results in loss of function and poor prognosis. AT-interacting domain-rich protein 1A (ARID1A), a key noncatalytic subunit of SWI/SNF complexes, demonstrates one of the highest mutational prevalence across human malignancies, resulting in loss of protein expression (Halaburkova et al., 2020). Alterations in ARID1A play an important role in enhancing cell stemness, expediting cell-cycle progression, promoting EMT process, and inducing metabolic reprogramming (Wang et al., 2022; Mullen et al., 2021). Emerging evidence substantiates significant correlations between ARID1A deficiency and poor prognosis, with high risk of recurrence, progression and mortality (Zhang et al., 2023). Furthermore, several studies have revealed that alterations in SWI/SNF complex enhance immune checkpoint inhibitors (ICIs) (Wang et al., 2023) while inducing chemotherapeutic resistance (Xue et al., 2021; Huang et al., 2024). However, the effects of SWI/SNF subunit mutations on the sensitivity to EGFR-TKIs remain underexplored in current oncologic research.

In this study, we evaluated the roles of SWI/SNF complex subunit dysregulation in regulating EGFR-TKI responses. We found that the comutation or low expression of ARID1A attenuates the response to EGFR-TKIs in EGFR-mutant LUAD. These findings may provide new insights for improving EGFR-TKI efficacy and overcoming EGFR-TKI resistance in LUAD.

## 2 Materials and methods

### 2.1 Bioinformatics analysis

The genomic data of 12862 LUAD patients from four large studies (MSK-CHORD, MSK-IMPACT Clinical Sequencing Cohort, China Pancancer, and MSK MetTropism) were extracted from cBioPortal (https://www.cbioportal.org/) (de Bruijn et al., 2023), which were used to analyse the frequencies of EGFR and SWI/SNF family member mutations in LUAD. Due to the lack of survival data from the China Pancancer study, we selected 3245 LUAD patients with EGFR mutations from three studies (MSK-CHORD, MSK-IMPACT Clinical Sequencing Cohort, and MSK MetTropism) for further survival analysis.

### 2.2 Clinical study design and patients

This single-center cohort analysis evaluated LUAD cases harboring classical EGFR alterations (exon19del/L858R) who underwent curative resection at Qingdao University Affiliated Hospital (01/2018-01/2020), with the follow-up ending on 11/2024. Eligibility required: (i) histologically confirmed EGFR-driven LUAD; (ii) ECOG-PS 0-2; (iii) complete TKI treatment records. Exclusion involved: (i) major organ dysfunction (cardiac/hepatic/renal); (ii) concurrent non-NSCLC malignancies; (iii) incomplete molecular profiling or off-protocol therapies.

The study was divided into 2 cohorts based on the treatment protocols. Cohort A consisted of 30 patients who received EGFR-TKIs as a first-line treatment after postoperative progression. Clinicopathological profiles and molecular characteristics were systematically documented in Table 1. Cohort B included 77 patients who received postoperative adjuvant EGFR-TKIs. The clinical characteristics of these patients are shown in Table 2. ARID1A protein quantification utilized standardized immunohistochemistry, with cohort stratification according to validated IHC scoring thresholds. The study was approved by the Ethics Committee of the Affiliated Hospital of Qingdao University (NO. QYFY WZLL 29417).

**TABLE 1:** Baseline characteristics for patients receiving EGFR-TKIs as a first-line treatment after postoperative progression.

Characteristics	Total (N = 30)	*ARID1A* low expression (N = 11)	*ARID1A* high expression (N = 19)	*P*-value
Age, median (range)	61(43–76)	60(49–71)	63(43–76)	0.919
≤60	14(46.7)	5(45.5)	9(47.4)	
>60	16(53.3)	6(54.5)	10(52.6)	
Gender, n (%)				0.643
Male	12(40.0)	5(45.5)	7(36.8)	
Female	18(60.0)	6(54.5)	12(63.2)	
ECOG performance, n (%)				0.236
0–1	25(83.3)	8(72.7)	17(89.5)	
2	5(16.7)	3(27.3)	2(10.5)	
*EGFR* mutation, n (%)				0.858
*19del*	14(46.7)	6(54.5)	8(42.1)	
*21 L858R*	16(53.3)	5(45.5)	11(57.8)	
EGFR-TKIs, n (%)				0.491
Gefitinib	4(13.3)	1(9.1)	3(15.8)	
Icotinib	15(50.0)	4(36.3)	11(57.9)	
Afatinib	2(6.7)	1(9.1)	1(5.3)	
Osimertinib	9(30.0)	5(45.5)	4(21.0)	

**TABLE 2:** Baseline characteristics for patients receiving postoperative adjuvant EGFR-TKIs treatments.

Characteristics	Total	*ARID1A* low expression	*ARID1A* high expression	*P-*value
	(N = 77)	(N = 21)	(N = 56)	
Age, median (range)	61(33–77)	58(38–72)	62(33–77)	0.301
≤60	33(42.9)	11(52.4)	22(39.3)	
>60	44(57.1)	10(47.6)	34(60.7)	
Gender, n (%)				0.961
Male	26(33.8)	7(33.3)	19(33.9)	
Female	51(66.2)	14(66.7)	37(66.1)	
ECOG performance, n (%)				0.936
0–1	70(90.9)	19(90.5)	51(91.1)	
2	7(9.1)	2(9.5)	5(8.9)	
Stage, n (%)				0.460
I	12(15.6)	3(14.3)	9(16.1)	
II	22(28.6)	4(19.1)	18(32.1)	
III	43(55.8)	14(66.7)	29(51.8)	
T- Stage, n (%)				0.217
T1	25(32.5)	5(23.8)	20(35.8)	
T2	38(49.3)	10(47.6)	28(50.0)	
T3	5(6.5)	1(4.8)	4(7.1)	
T4	9(11.7)	5(23.8)	4(7.1)	
N- Stage, n (%)				0.998
N0	22(28.6)	6(28.6)	16(28.6)	
N1	18(23.4)	5(23.8)	13(23.2)	
N2	37(48.0)	10(47.6)	27(48.2)	
*EGFR* mutation, n (%)				0.226
*19del*	39(50.6)	13(61.9)	26(46.4)	
*21 L858R*	38(49.4)	8(38.1)	30(53.6)	
EGFR-TKIs, n (%)				0.332
Gefitinib	20(26.0)	6(28.6)	14(25.0)	
Icotinib	30(39.0)	5(23.8)	25(44.7)	
Afatinib	7(9.1)	2(9.5)	5(8.9)	
Osimertinib	20(25.9)	8(38.1)	12(21.4)	

### 2.3 Immunohistochemical staining

**FIGURE 1:**
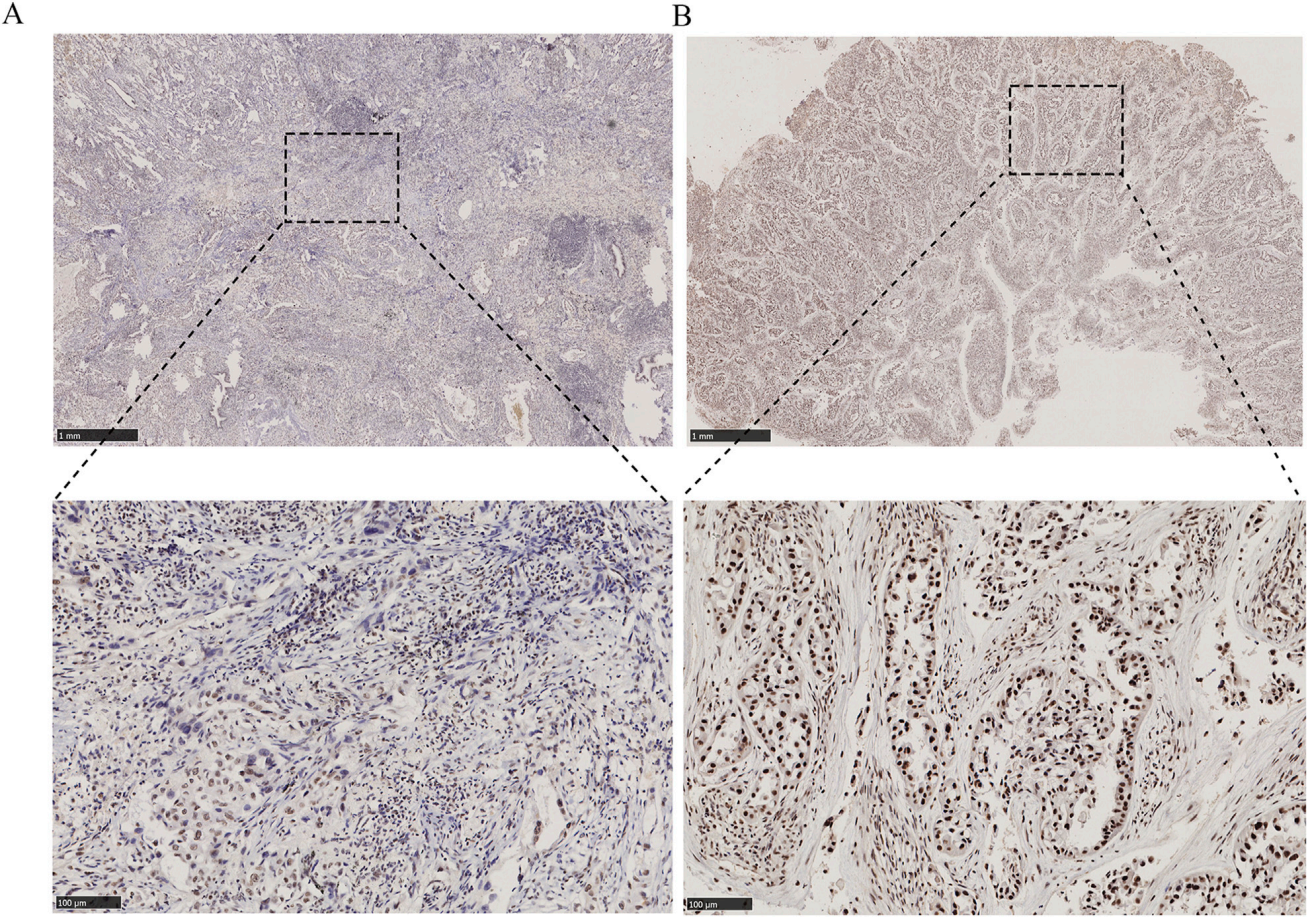
Immunohistochemical staining for ARID1A expression in EGFR-mutant LUAD tissues (50×and 200×). **(A)** ARID1A low expression. **(B)** ARID1A high expression.

Histological specimens were processed through sequential dewaxing in xylene, graded alcohol rehydration, and Tris-EDTA-mediated epitope unmasking via microwave irradiation, followed by BSA blocking. Immunodetection involved overnight 4°C incubation with anti-ARID1A primary antibody (Proteintech, 1:500) and subsequent HRP-conjugated secondary antibody for 60 min. Chromogenic development utilized DAB substrate under standardized conditions. Blinded histopathological assessment by two board-certified pathologists employed. Nuclear staining intensity: 0 = negative, 1 = weak, 2 = moderate, 3 = strong. Positive cellular prevalence: 0 (≤5%), 1 (6%–25%), 2 (26%–50%), 3 (51%–75%), and 4 (>75%). The score of each section (range 0–12) was calculated by multiplying nuclear staining intensity with positive cellular prevalence. Specimens were stratified into ARID1A low-expression (score <6) and high-expression (score≥6) cohorts based on median composite scores. Representative IHC patterns shown in Figures 1A, B.

### 2.4 Construction of a nomogram

A multivariate Cox regression-derived prognostic model incorporating ARID1A quantification and key clinicopathological variables was developed using the R “rms” module. A time-dependent calibration curve was employed to verify survival estimation precision.

### 2.5 Cell culture and transfection

LUAD cell line PC-9 (Procell Life Science, Wuhan, China) was maintained in RPMI-1640 medium supplemented with 10% FBS under standard humidified conditions (37°C, 5% CO_2_). For stable genetic modification, lentiviral vectors encoding ARID1A-specific shRNA (GeneChem, Shanghai, China) were transduced according to the manufacturer’s instructions. Sequences of shRNA was shown as follows: shARID1A, TTC​TCC​GAA​CGT​GTC​ACG​T.

### 2.6 CCK8 assay

After 24 h incubation in 96-well microplates (5 × 10^3^ cells/well), cellular viability was assessed through osimertinib dose-response profiling (0–10μM; Selleck Chemicals). 24 h later, cell proliferation was quantified using CCK-8 assay (TargetMol) with 450 nm optical density measurements via spectrophotometry.

### 2.7 Flow cytometry analysis

Following experimental-specific interventions in 6-well culture systems, cellular apoptosis was analysed using Annexin V-647/PI apoptosis detection assay following manufacturer protocols (Life-iLab, China).

### 2.8 Transwell migration assay

Serum-deprived cell suspensions (2 × 10^4^ cells) were seeded into upper compartments with RPMI-1640 medium, while the lower chamber contained 10% FBS as chemoattractant. After 24 h culture, membranes with migratory cells were processed through sequential immobilization (4% paraformaldehyde, 30min) and nuclear counterstaining (0.1% crystal violet, 20min).

### 2.9 Western blotting (WB)

Cellular protein lysates were prepared with RIPA lysis buffer (Beyotime) containing protease/phosphatase inhibitors (MedChemExpress). Electrophoretic separation (8%–10% SDS-PAGE) preceded nitrocellulose membrane transfer. Post-blocking with 5% skim milk/TBST, blots were probed with primary antibodies (4°C, overnight) followed by HRP-secondary antibodies incubation (room temperature, 2 h). Chemiluminescent visualization utilized ECL detection. Primary antibodies specifications are provided in Supplementary material.

### 2.10 Gene set enrichment analysis (GSEA) of ARID1A

To delineate the functions of ARID1A, we performed functional analysis of ARID1A via the LinkedOmics database (https://www.linkedomics.org/login.php) (Vasaikar et al., 2018). ARID1A-related coexpressed genes were screened via the Pearson correlation test. The GSEA method was subsequently selected and used for GO and KEGG analyses of coexpressed genes.

### 2.11 Statistical analysis

Statistical processing utilized GraphPad Prism 8.0 and SPSS 25.0 analytical software. Intergroup comparisons employed T tests and chi-square tests. Survival data was evaluated through Kaplan-Meier curve modeling with log-rank validation. *P* < 0.05 was considered to indicate statistical significance.

## 3 Results

### 3.1 The roles of concurrent SWI/SNF subunit mutations in EGFR-mutant LUAD

**FIGURE 2:**
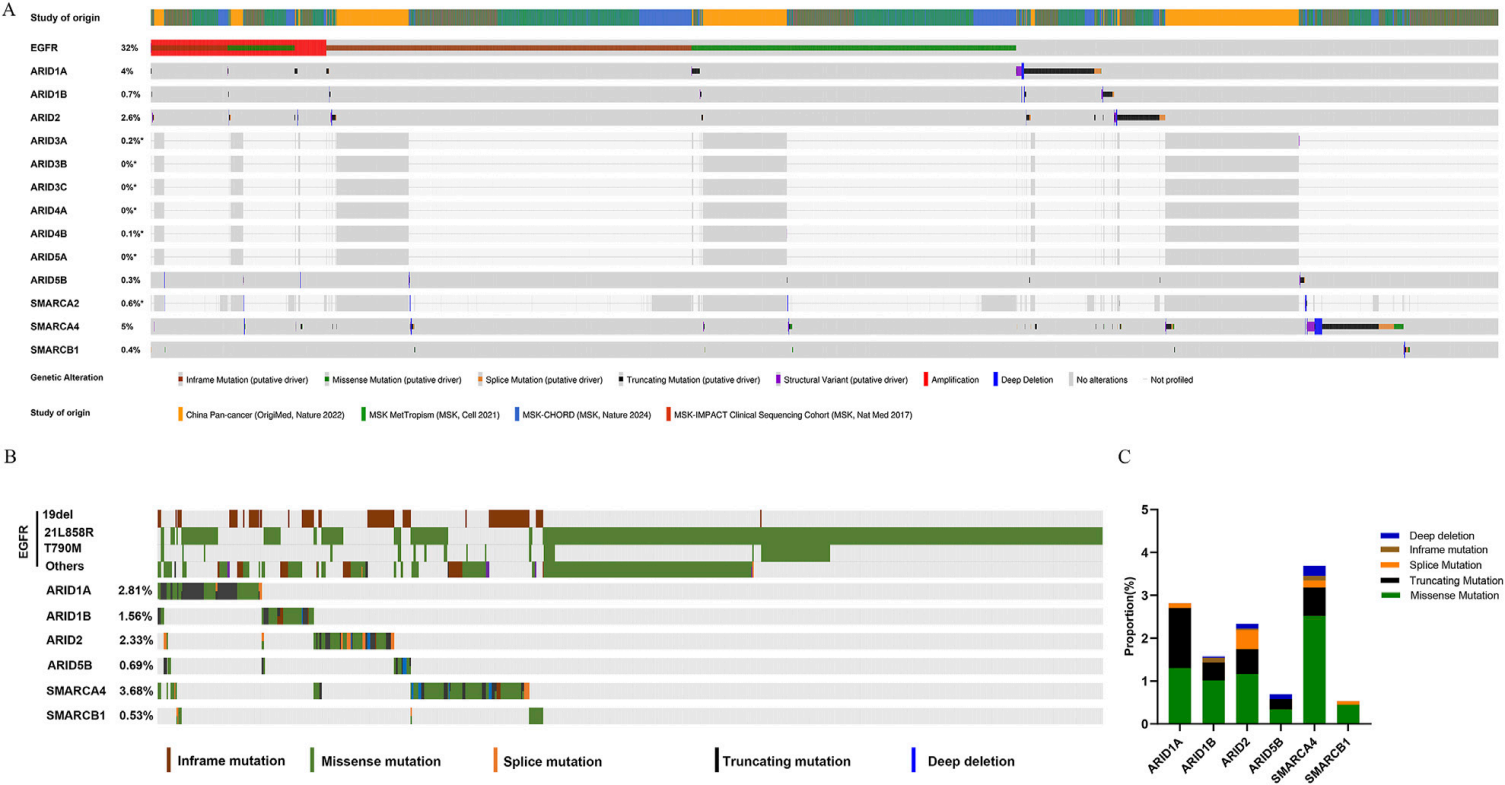
SWI/SNF subunit mutations in lung adenocarcinoma (LUAD). **(A)** The frequencies of EGFR and SWI/SNF subunit mutations in LUAD. **(B)** The frequency of SWI/SNF subunit mutations in EGFR-mutant LUAD. **(C)** The types of SWI/SNF subunit mutations in EGFR-mutant LUAD.

Based on genomic data from the cBioportal database, analyses of the mutation frequencies of EGFR and SWI/SNF subunits revealed that EGFR (32%), ARID1A (4%), ARID1B (0.7%), ARID2 (2.6%), ARID3A (0.2%), ARID4B (0.1%), ARID5B (0.3%), SMARCA2 (0.6%), SMARCA4 (5%) and SMARCB1 (0.4%) mutations were detected, whereas ARID3B, ARID3C, ARID4A and ARID5A gene mutations were not detected in LUAD (Figure 2A). We also explored the mutation frequencies of concurrent SWI/SNF subunit mutations in EGFR-mutant LUAD. As shown in Figure 2B, the mutation frequencies of the ARD1A, ARID1B, ARID2, ARID5B, SMARCA4 and SMARCB1 genes were 2.81%, 1.56%, 2.33%, 0.69%, 3.68% and 0.53%, respectively, and these mutations mainly involved missense mutations and truncating mutations in EGFR-mutant LUAD (Figure 2C).

**FIGURE 3:**
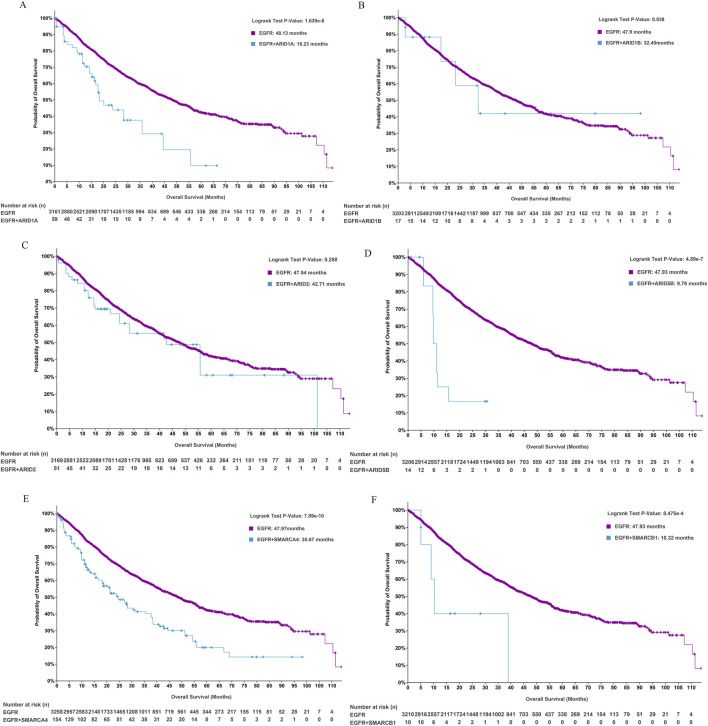
The role of SWI/SNF subunit mutations in the prognosis of EGFR-mutant LUAD. **(A-F)** ARID1A, ARID1B, ARID2, ARID5B, SMARCA4, and SMARCB1.

We further analysed whether concurrent SWI/SNF subunit alterations affected the prognosis of patients with EGFR-mutant LUAD. Patients with alterations in ARID1A (18.23 versus 48.13 months, *P* < 0.0001, Figure 3A), ARID5B (9.76 versus 47.93 months, *P* < 0.0001, Figure 3D), SMARCA4 (30.67 versus 47.97 months, *P* = 0.0001, Figure 3E) and SMARCB1 (10.32 versus 47.93 months, *P* = 0.0008, Figure 3F) had worse OS than patients with wild-type genes in EGFR-mutant LUAD, and gene alterations in ARID1B (32.45 versus 47.9 months, *P* = 0.838) and ARID2 (42.71 versus 47.54 months, *P* = 0.258) exerted no effect on the OS of patients with EGFR-mutant LUAD. ARID1A, which is a key subunit of SWI/SNF complexes, plays an essential role in preventing oncogene-driven tumorigenesis as a tumour suppressor gene (Mullen et al., 2021). We subsequently focused on the prognostic effect of ARID1A/EGFR comutation in LUAD.

### 3.2 ARID1A/EGFR comutation is associated with a poor prognosis in patients with EGFR-mutant LUAD

**FIGURE 4:**
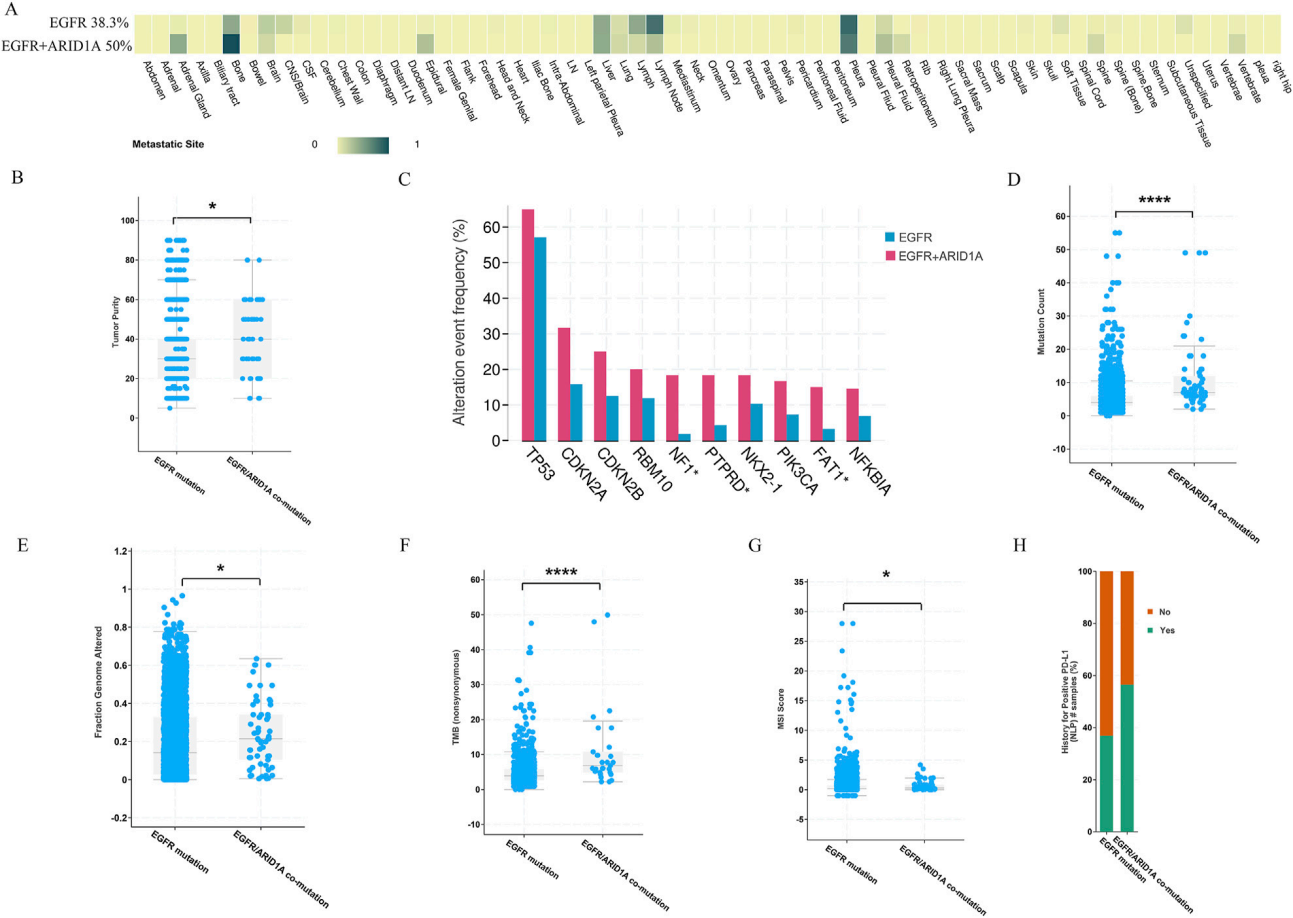
Comparison of clinical features between patients with ARID1A mutations and patients with wild-type ARID1A. **(A)** The number of patients with distant metastases. **(B)** Tumour purity. **(C)** The frequency of gene alterations. **(D)** Mutation count. **(E)** Fractions of genome alterations. **(F)** TMB. **(G)** MSI score. **(H)** The rate of PDL1 positivity. *P < 0.05, ****P < 0.0001.

To further evaluate the prognostic value of ARID1A mutations in EGFR-mutant LUAD, we assessed the clinical characteristics of patients and revealed that patients with ARID1A/EGFR comutations were more likely to develop distant metastases. Patients with ARID1A mutations tended to exhibit increased rates of bone and pleural metastases, whereas patients with wild-type ARID1A predominantly exhibited lymph node and pleural metastases (Figure 4A). The tumour purity of the ARID1A mutant group was greater than that of the wild-type group (Figure 4B). The prevalence of comutations in the ARID1A-mutant group was also greater than that in the ARID1A-wild-type group, and TP53 alterations were the most common co-occurring mutations (65%), followed by CDKN2A alterations (31.67%) (Figure 4C). We also observed the same trends in mutation count (7 versus 4, *P* < 10^-10^, Figure 4D), fraction of genome alterations (21% versus 14%, *P* = 0.01, Figure 4E), TMB (6.71 versus 3.46, *P* < 10^-10^, Figure 4F), MSI score (0.29 versus 0.16, *P* = 0.01, Figure 4G) and the rate of PDL1 positivity (56.52% versus 36.91%, Figure 4H). Current oncogenomic evidence demonstrates that elevated PD-L1 expression and TMB correlate with diminished EGFR-TKI efficacy in EGFR-driven LUAD (Ding et al., 2024; Offin et al., 2019). These findings further indicate co-occurring ARID1A/EGFR genomic alterations as novel biomarkers for adverse survival outcomes in LUAD.

**FIGURE 5:**
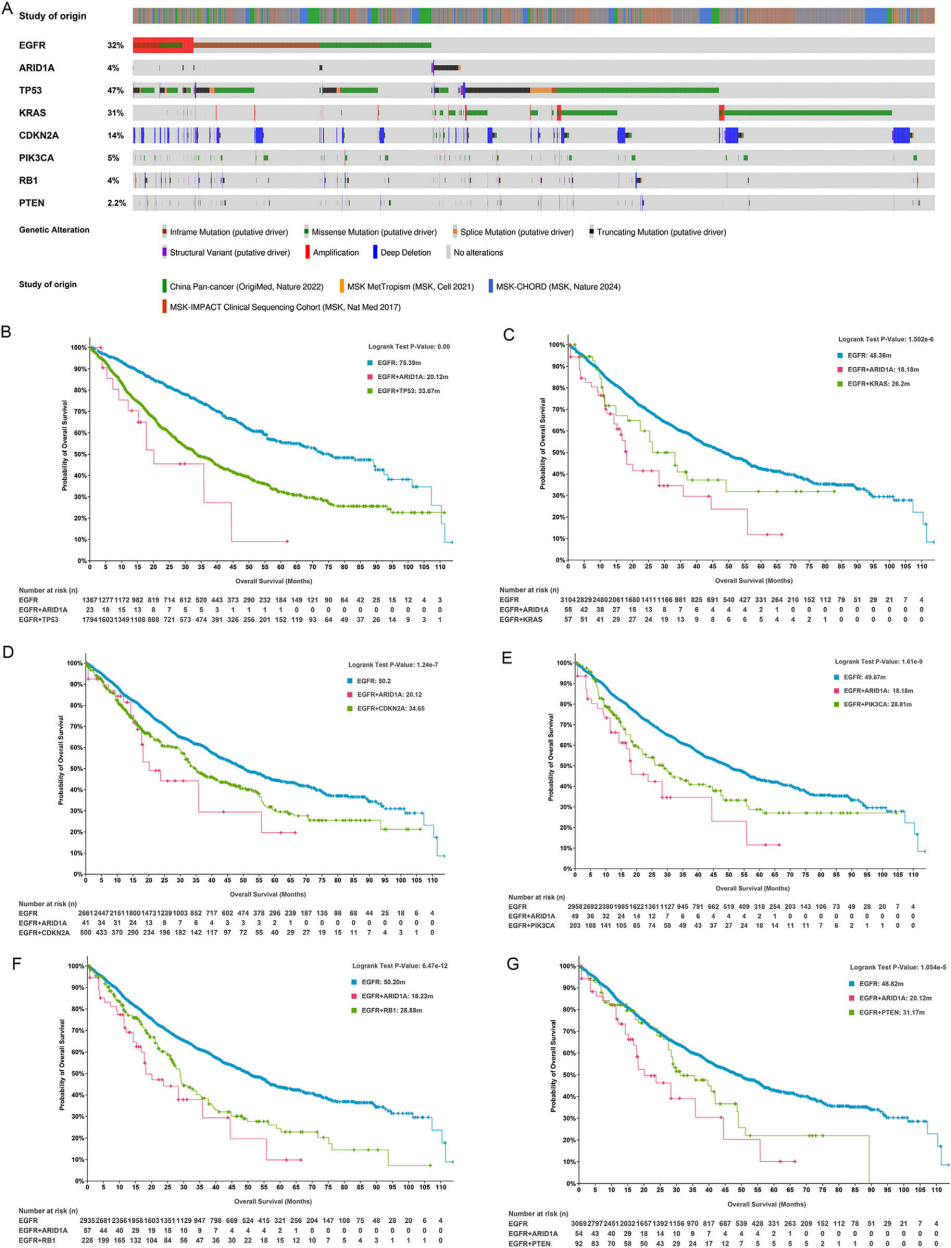
ARID1A mutation confers a poor prognosis for patients with EGFR-mutant LUAD. **(A)** The frequencies of genes exhibiting coexisting mutations with EGFR. Survival analysis for patients with the EGFR mutation, ARID1A/EGFR comutation and **(B)** TP53/EGFR, **(C)** KRAS/EGFR, **(D)** CDKN2A/EGFR, **(E)** PIK3CA/EGFR, **(F)** RB1/EGFR, and **(G)** PTEN/EGFR comutations.

Rapid advances in genomics have revealed that concurrent genetic alterations are correlated with poor prognosis in patients with EGFR-mutant LUAD. Currently, known concurrent genetic alterations that weaken the efficacy of EGFR-TKIs include TP53, PIK3CA, PTEN, KRAS, RB1, and CDKN2A (Sun et al., 2022; Hellyer et al., 2022). TP53 alterations predominated in the genomic landscape, detected in 47% of analyzed specimens, followed by mutations in KRAS (31%), CDKN2A (14%), PIK3CA (5%), RB1 (4%), and PTEN (2.2%) in LUAD (Figure 5A). We compared the effects of ARID1A and the abovementioned six genes coexisting with EGFR mutations on the prognosis of patients with EGFR-mutant LUAD. As illustrated in Figure 5B, ARID1A/EGFR and TP53/EGFR comutations were both associated with poor prognoses in patients with LUAD, and the comutation of ARID1A was associated with worse OS than TP53 (EGFR: 75.39 months; ARID1A/EGFR comutation: 20.12 months; TP53/EGFR comutation: 33.67 months; *P* < 0.01). These trends were also observed in KRAS (EGFR: 48.36 months; ARID1A/EGFR comutation: 18.18 months; KRAS/EGFR comutation: 26.2 months; *P* < 0.0001, Figure 5C), CDKN2A (EGFR: 50.2 months; ARID1A/EGFR comutation: 20.12 months; CDKN2A/EGFR comutation: 34.65 months; *P* < 0.0001, Figure 5D), PIK3CA (EGFR: 49.87 months; ARID1A/EGFR comutation: 18.18 months; PIK3CA/EGFR comutation: 28.81 months; *P* < 0.0001, Figure 5E), RB1 (EGFR: 50.2 months; ARID1A/EGFR comutation: 18.23 months; RB1/EGFR comutation: 28.88 months; *P* < 0.0001, Figure 5F) and PTEN mutations (EGFR: 48.82 months; ARID1A/EGFR comutation: 20.12 months; PTEN/EGFR comutation: 31.17 months; *P* < 0.0001, Figure 5G). Thus, the ARID1A mutation was associated with poor OS in EGFR-mutant LUAD patients, and the prognostic influence was greater than that of concurrent EGFR mutations in TP53, KRAS, CDKN2A, PIK3CA, RB1 or PTEN.

### 3.3 Low ARID1A expression is associated with poor prognosis in patients receiving EGFR-TKIs as the first-line treatment after postoperative progression in EGFR-mutant LUAD

**FIGURE 6:**
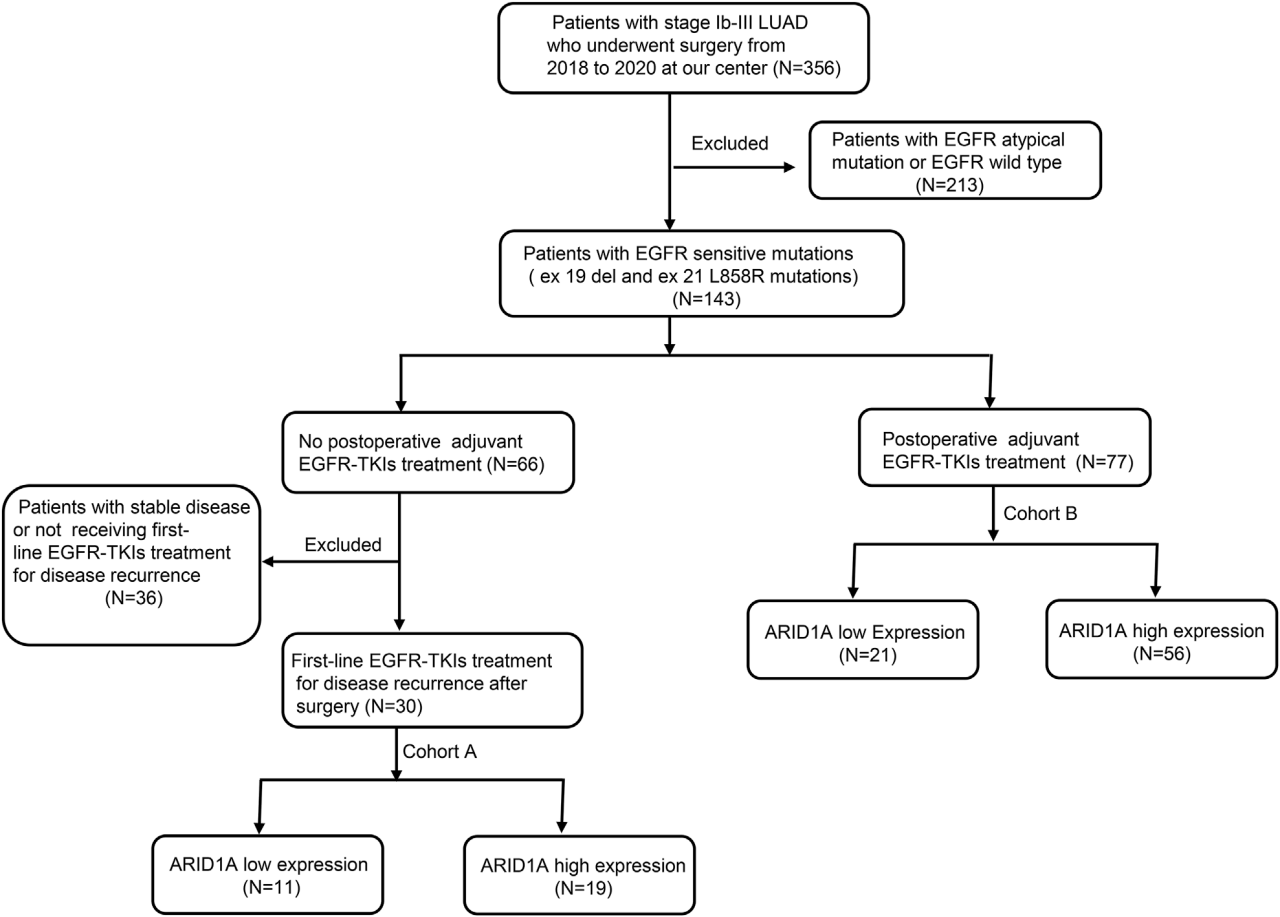
Flowchart of clinical study in our centre.

**FIGURE 7:**
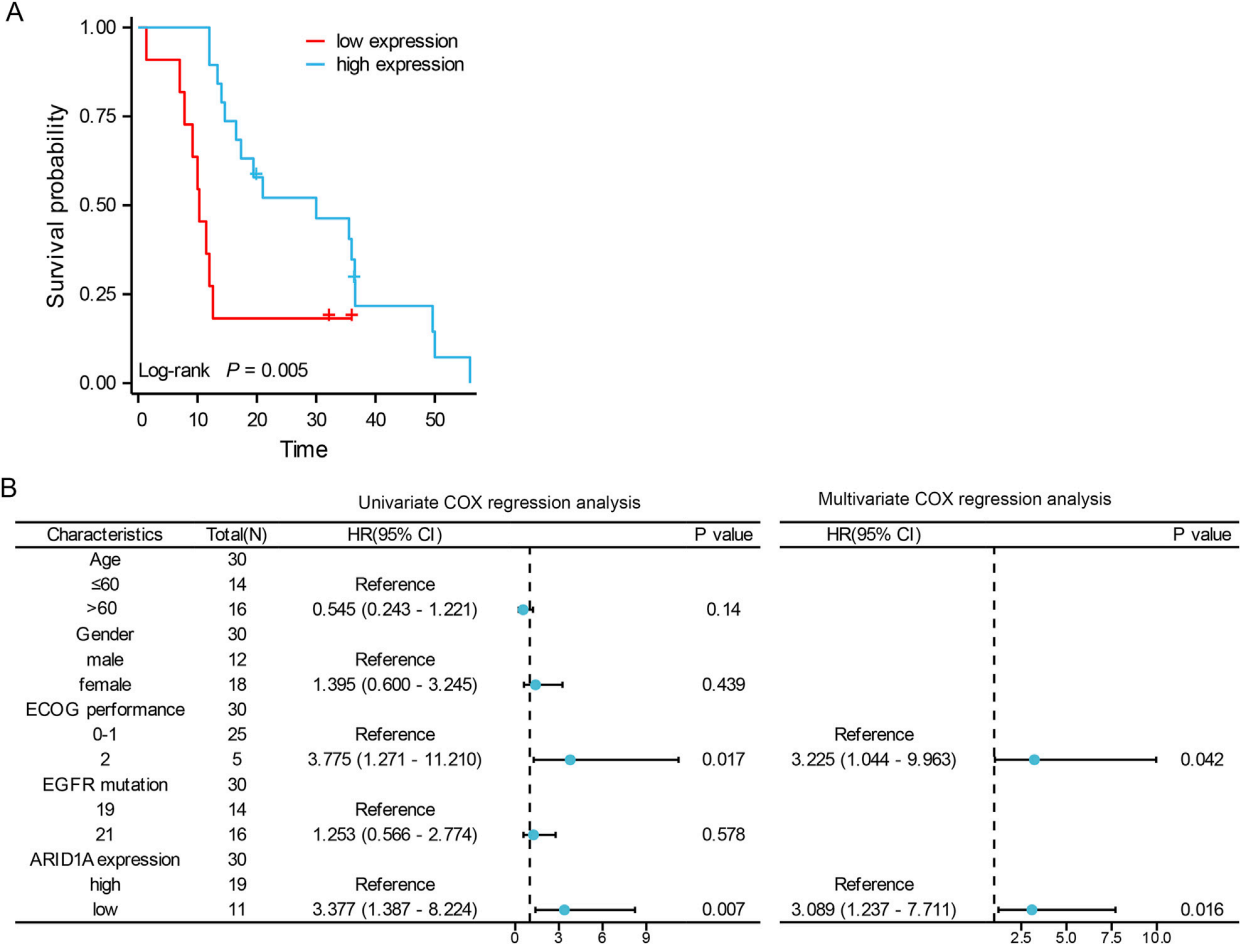
The role of ARID1A expression in the prognosis of EGFR-mutant LUAD patients receiving EGFR-TKIs as a first-line treatment after postoperative progression. **(A)** Comparison of PFS between patients with low ARID1A expression and patients with high ARID1A expression. **(B)** Forest plots for the univariate and multivariate analyses of PFS.

A total of 30 patients who received treatment with EGFR-TKIs as the first-line treatment after postoperative progression were enrolled in cohort A (Figure 6). As shown in Table 1, stratification by ARID1A expression levels revealed 11 cases with low expression and 19 cases with high expression. Significantly, shorter median PFS was observed in the ARID1A-low subgroup (10.3 vs. 30 months; HR = 2.879, 95%CI 0.996-8.320, *P* = 0.005; Figure 7A). Univariate Cox modeling identified low ARID1A expression (HR = 3.377, 95%CI 1.387-8.224, *P* = 0.007) and an ECOG performance status (PS) of 2 (HR = 3.775, 95%CI 1.271-11.210, *P* = 0.017) as significant predictors of shorter PFS (Figure 7B). Multivariable analysis confirmed both ARID1A deficiency (HR = 3.089, 95%CI 1.237-7.711, *P* = 0.016) and an ECOG-PS of 2 (HR = 3.225, 95%CI 1.044-9.963, *P* = 0.042) as independent prognostic markers. These findings indicate that loss of ARID1A expression attenuates the response to EGFR-TKIs in EGFR-mutant LUAD patients receiving a first-line treatment after postoperative progression.

### 3.4 Loss of ARID1A expression predicts a poor prognosis in patients receiving postoperative adjuvant EGFR-TKI treatment in EGFR-mutant LUAD

**FIGURE 8:**
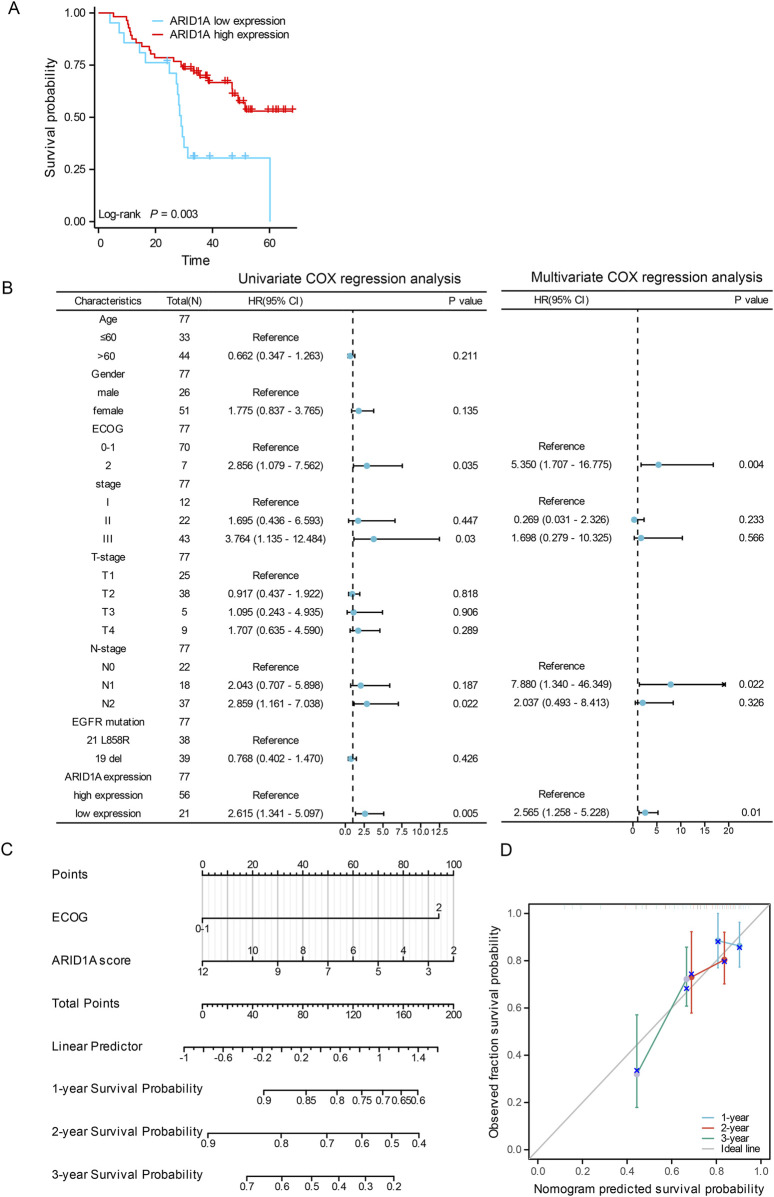
The role of ARID1A in the prognosis of EGFR-mutant LUAD patients receiving postoperative adjuvant EGFR-TKI treatments. **(A)** Comparison of DFS between patients with low ARID1A expression and patients with high ARID1A expression. **(B)** Forest plots for univariate and multivariate analyses of DFS. **(C)** Nomogram for the prediction of 1-, 2- and 3-year survival. **(D)** Calibration curves of the nomogram.

Cohort B comprised 77 EGFR-mutant LUAD cases undergoing postoperative adjuvant EGFR-TKIs therapy (Figure 6). ARID1A expression stratification identified 21 cases with low expression and 56 cases with high expression (Table 2). Survival analysis demonstrated significantly reduced median DFS in ARID1A-low subgroup (29 months, 95%CI 27.33-NA) compared to ARID1A-high subgroup (NA, 95%CI 47-NA; *P* = 0.003) (Figure 8A).Univariate Cox modeling revealed four adverse prognostic factors: ARID1A deficiency (HR = 2.615, 95%CI 1.341-5.097, *P* = 0.005), an ECOG-PS of 2 (HR = 2.856, 95%CI 1.079-7.562, *P* = 0.035), Stage III (HR = 3.764, 95%CI 1.135-12.484, *P* = 0.030), and N2 lymph node metastasis (HR = 2.859, 95%CI 1.611-7.038, *P* = 0.022). Subsequent multivariate analysis confirmed ARID1A deficiency (HR = 2.565, 95%CI 1.258-5.228, *P* = 0.010) and ECOG-PS of 2 (HR = 5.350, 95%CI 1.707-16.775, *P* = 0.004) as independent DFS predictors (Figure 8B). A prognostic nomogram integrating ARID1A score and ECOG-PS was constructed to predict the DFS of patients (Figure 8C), validated through the calibration curves (Figure 8D). These findings define ARID1A deficiency as a key mediator of EGFR-TKI resistance in EGFR-mutant LUAD patients receiving postoperative adjuvant EGFR-TKI treatments.

### 3.5 Loss of ARID1A expression confers resistance to osimertinib *in vitro*

**FIGURE 9:**
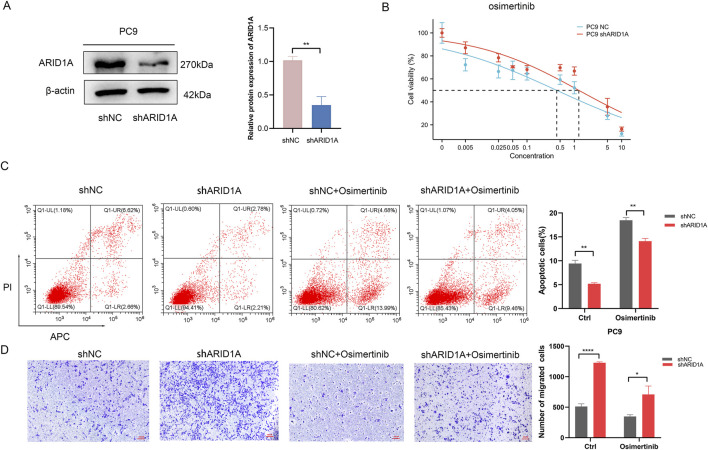
Loss of ARID1A expression confers resistance to Osimertinib in PC9 cell. **(A)** Western blot analysis of ARID1A protein expression in the control group (shNC) and the knockdown group (shARID1A). **(B)** The IC50 for osimertinib in the control group and the knockdown group. **(C)** Flow cytometry analysis of ARID1A konckdown effect on apoptosis with and without osimertinib treatment. **(D)** Transwell migration analysis of ARID1A konckdown effect on migration with and without osimertinib treatment. ^* *^ *P* < 0.01, ^****^ *P* < 0.0001.

In order to elucidate the effects of ARID1A deficiency on the efficacy of EGFR-TKI, we employed a lentivirus to construct the ARID1A knockdown model in EGFR-sensitive mutated PC9 cell. ARID1A knockdown efficiency was verified by WB (Figure 9A). We calculated the IC50 for osimertinib in PC9 cell, the results revealed that ARID1A knockdown led to a threefold increase in the IC50 value of osimertinib when compared to the control group (Figure 9B). Flow cytometry showed that ARID1A knockdown caused inhibition of cell apoptosis and pro-apoptotic effects of osimertinib (Figure 9C). Transwell migration assay demonstrated that ARID1A knockdown promoted cell migration and inhibited the anti-tumor migration ability of osimertinib (Figure 9D). These results indicate that loss of ARID1A expression attenuates the efficacy of osimertinib in PC9 cell.

**FIGURE 10:**
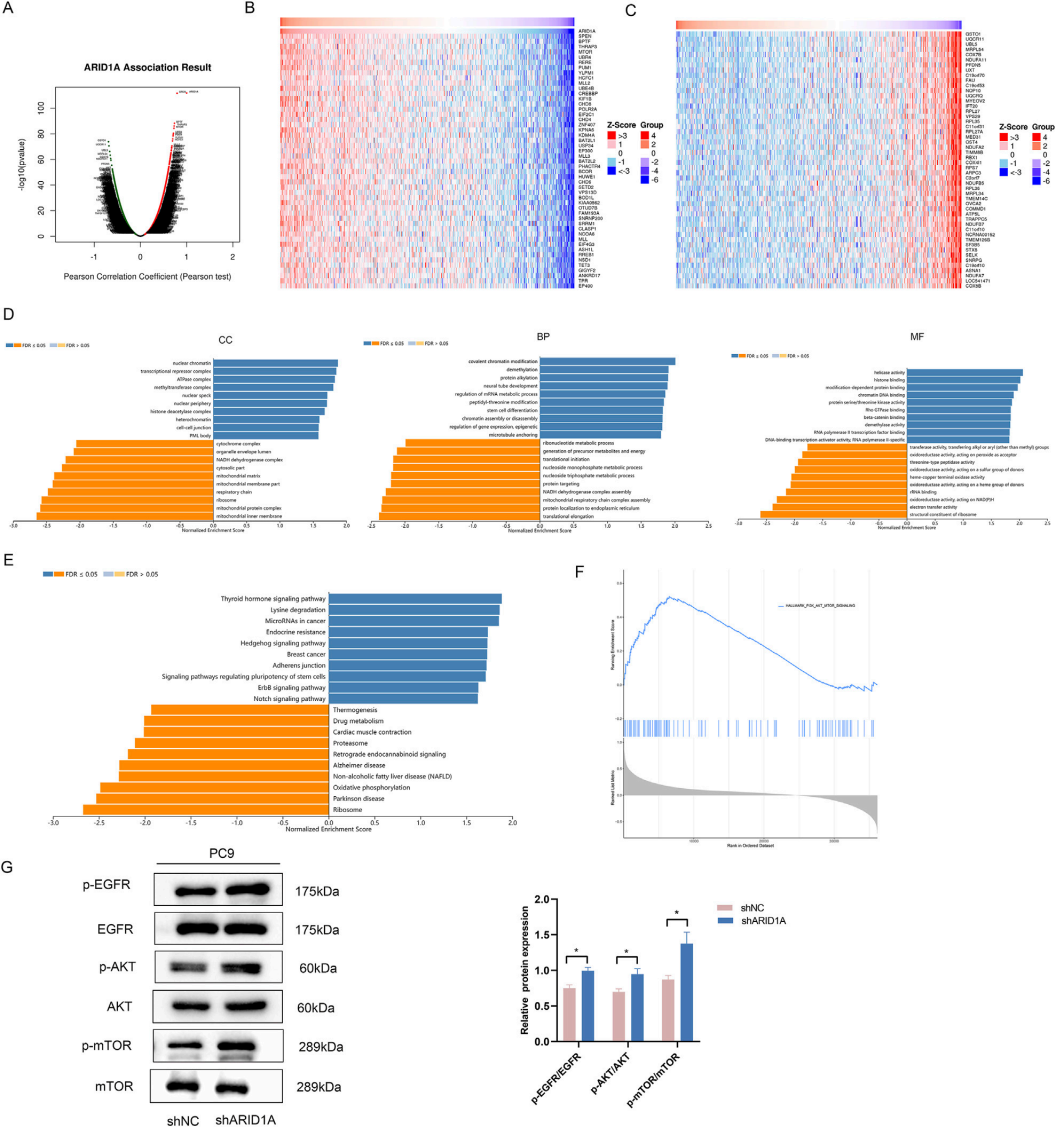
ARID1A knockdown confers resistance to EGFR-TKI by activating the EGFR/AKT/mTOR signalling pathway. **(A)** Genes coexpressed with ARID1A. **(B)** The top 50 genes that were positively associated with ARID1A. **(C)** The top 50 genes that were negatively associated with ARID1A. **(D)** GO analysis of ARID1A coexpressed genes. CC: cellular component, BP: biological process, MF: molecular function. **(E)** KEGG analysis of ARID1A coexpressed genes. **(F)** GSEA analysis of ARID1A via TCGA database. **(G)** Western blot analysis of ARID1A konckdown effect on EGFR/AKT/mTOR pathway-related protein. ^*^ *P* < 0.05.

To explore the potential mechanism of ARID1A, we performed functional analysis of ARID1A via the LinkedOmics database. We firstly analysed the genes coexpressed with ARID1A (Figure 10A) and used heatmaps to visualize the top 50 genes that were positively and negatively associated with ARID1A (Figures 10B, C). GSEA was subsequently performed on the top 100 genes coexpressed with ARID1A. The results of the GO analysis revealed a significant function in the regulation of chromatin remodelling and gene expression (Figure 10D). The results of the KEGG analysis were mainly enriched in the Hedgehog signalling pathway, regulation of stem cells, ErbB signalling pathway, Notch signalling pathway and oxidative phosphorylation (Figure 10E). GSEA analysis of ARID1A was also conducted via TCGA database. The results showed that enrichment of ARID1A was involved in the PI3K/Akt/mTOR signalling pathway (Figure 10F). Studies have shown that abnormal activation of the ErbB signalling pathway activates the downstream PI3K/Akt/mTOR pathway, which are the key EGFR-independent signalling pathways that cause resistance to EGFR-TKIs (Liu et al., 2018). We further detected the effects of knockdown ARID1A on the EGFR/AKT/mTOR pathway using WB. As shown in Figure 10G, ARID1A knockdown activated the EGFR/AKT/mTOR signalling pathway by promoting expression of p-EGFR, p-AKT and p-mTOR. Thus, ARID1A knockdown confers resistance to osimertinib by activating the EGFR/AKT/mTOR signalling pathway.

## 4 Discussion

Although the majority of EGFR-mutant NSCLC patients benefit from treatment with EGFR-TKIs, acquired resistance is inevitable, thus leading to tumor recurrence and metastasis. Therefore, the identification of novel candidate targets to clarify the mechanism of EGFR-TKI resistance, predict patient response and optimize patient selection is highly important. ARID1A, which encodes a key subunit of the SWI/SNF complex, plays a critical role in regulating gene expression by controlling chromatin accessibility and histone acetylation (Zhang et al., 2023). ARID1A alterations occur across many cancers, accounting for approximately 5%–10% of NSCLC. Sun et al. found that ARID1A deficiency drove metastatic progression in LUAD (Sun et al., 2021). Huang et al. revealed that loss of ARID1A upregulated VASN expression via the NOTCH1 signalling pathway, contributing to lung cancer advancement (Wu et al., 2024). ARID1A deficiency due to somatic mutations is an independent prognostic factor for poor OS in patients with NSCLC (Hung et al., 2020). In this study, we explored the roles of ARID1A mutations in EGFR-mutant LUAD and concluded that ARID1A mutations are associated with significantly poor OS and a greater proportion of distant metastases in EGFR-mutant LUAD patients.

Emerging evidence highlights TMB and PD-L1 overexpression as dual biomarkers predictive of ICIs response, with growing attention to TKI therapeutic resistance. Current studies have revealed that TMB is increased in TKI-resistant patients and correlate with reduced OS in EGFR-driven LUAD (Offin et al., 2019). Similarly, high PDL1 expression has been detected in EGFR-TKI-resistant samples and shown to be correlated with primary resistance to EGFR-TKIs (Hsu et al., 2019). In our study, a high PD-L1 positive rate and TMB were observed in patients with ARID1A mutations, thereby suggesting that ARID1A mutations indicate a poor response to EGFR-TKI treatment and a poor prognosis in patients with LUAD. Furthermore, studies have shown that concurrent genetic alterations and genomic instability play major roles in cancer heterogeneity and drug resistance (Skoulidis and Heymach, 2019). Chen et al. (Guo et al., 2020) found that concurrent genetic alterations were negatively correlated with ORR, PFS and drug responses in patients with EGFR-mutant LUAD treated with EGFR-TKIs as a first-line therapy. Chang et al. (Chang et al., 2019) reported that patients with any concomitant mutations exhibited a worse OS when treated with first-generation EGFR-TKIs. Moreover, TP53 mutations have been identified as the most common concurrent mutations, with 30%–65% of patients exhibiting EGFR-mutant NSCLC (Hou et al., 2019). TP53 mutations are associated with a poor prognosis even in patients with a good response to initial EGFR-TKI treatment and lead to rapid acquired resistance by initiating genetic instability and mutagenicity (Vokes et al., 2022). Other comutations that have been identified as independent prognostic markers for poor prognosis include KRAS, CDKN2A, PIK3CA, RB1 and PTEN mutations (Sun et al., 2022; Hellyer et al., 2022). In our study, we compared the effects of ARID1A and the abovementioned six genes coexisting with EGFR mutations on the prognosis of patients with EGFR-mutant LUAD. The results revealed that the ARID1A comutation subgroup exhibited a worse OS than the comutation subgroup with the abovementioned six genes in EGFR-mutant LUAD. Therefore, the prognostic influence of ARID1A was greater than that of concurrent EGFR mutations in TP53, KRAS, CDKN2A, PIK3CA, RB1 or PTEN.

Many studies have shown that ARID1A serves as a potential predictive biomarker for cancer treatment. ARID1A deficiency potentiates immunotherapy efficacy by elevating PD-L1 expression and TMB, impairing mismatch repair (MMR) function, and remodelling the tumor immune microenvironment (TIME) (Wang et al., 2020). Loss of ARID1A expression also leads to chemotherapy resistance in a variety of tumors, including lung cancer (Huang et al., 2024), ovarian cancer (Duska et al., 2023) and pancreatic cancer (Li et al., 2022). There are few studies on the prognostic effect of ARID1A on EGFR-TKI. The current study only shows that advanced LUAD patients with low ARID1A expression exhibited worse PFS when receiving first-line treatment with first-generation EGFR-TKIs (Sun et al., 2023). To further investigate the impact of ARID1A on EGFR-TKI efficacy, particularly given that the incidence of the ARID1A comutation in EGFR-mutant LUAD is relatively low, and loss-of-function (LOF) alterations in ARID1A are likely to cause loss of protein expression (Jin et al., 2023). We established two clinical cohorts to analyse the effect of ARID1A expression on the response to EGFR-TKIs. We found that loss of ARID1A expression was an independent predictor of poor clinical prognosis in EGFR-mutant LUAD patients for whom treatment with EGFR-TKIs was used as a first-line treatment after postoperative progression (cohort A) or as a postoperative adjuvant therapy (cohort B). We also verified that ARID1A konckdown attenuated the efficacy of osimertinib in PC9 cell. These data suggest that ARID1A deficiency attenuates the response to EGFR-TKIs and is associated with poor prognosis in EGFR-mutant LUAD patients.

EGFR-TKI resistance mechanisms encompass secondary mutations (T790M/C797S), alternative pathway activation (MET/HER2 amplification), histological transformation, abnormal activation of downstream signalling pathway, and ABC transporter-mediated drug efflux (Sun et al., 2022; Dhanyamraju, 2024). In order to further explore the underlying mechanism by which the ARID1A deficiency attenuates the response to EGFR-TKIs in LUAD. We conducted the functional analysis of ARID1A via the LinkedOmics database. The results were mainly enriched in the Hedgehog signalling pathway, regulation of stem cells, ErbB signalling pathway, Notch signalling pathway and oxidative phosphorylation (OXPHOS). We also found that enrichment of ARID1A was involved in the PI3K/Akt/mTOR signalling pathway via TCGA database. EGFR (ErbB-1), which is a receptor tyrosine kinase (RTK) of the ErbB family, is activated by binding to ligands, after which it subsequently activates downstream pathways. Abnormal activation of the ErbB signalling pathway is involved in abnormal activation of the PI3K/Akt/PTEN/mTOR and RAS/RAF/MEK/ERK pathways, which are the key EGFR-independent signalling pathways that cause resistance to EGFR-TKIs (Liu et al., 2018). Our study verified that ARID1A knockdown activated the EGFR/AKT/mTOR signalling pathway by promoting expression of p-EGFR, p-AKT and p-mTOR in PC9 cell. Thus, ARID1A deficiency may confer resistance to EGFR-TKIs through aberrant activation of the EGFR-AKT-mTOR signalling axis.

The above results underscore the importance to optimize treatment for EGFR-driven LUAD characterized by concomitant ARID1A alterations. Metabolic reprogramming, which is a hallmark of cancer, contributes to acquired resistance, and the combination of an EGFR-TKI plus an inhibitor of OXPHOS reverses EGFR-TKI resistance (Lin et al., 2023). Emerging evidence highlights the PRC2 catalytic subunit EZH2 as a therapeutic dependency within ARID1A-deficient malignancies, and delineates a synthetic lethal interaction between ARID1A dysfunction and EZH2 inhibitors (Keller et al., 2024). Furthermore, ARID1A knockdown stimulates neovascularization through transcriptional modulation of Ang-2; conversely, anti-vascular therapy attenuates the invasive phenotypes of tumor (Yoodee et al., 2021). The abovementioned studies provide a basis for exploring a promising combination therapy strategy. Phosphatase inhibitors, EZH2 inhibitors, and anti-angiogenic treatment may synergize with EGFR-TKI therapy in EGFR-mutant LUAD characterized by ARID1A deficiency.

While this study validates ARID1A deficiency as a predictive biomarker for EGFR-TKI in LUAD, the constrained sample size inevitably reduced the universality of the study. Larger-scale clinical trials are essential to validate the efficacy and search for appropriate complementary therapies.

## 5 Conclusion

In conclusion, our study further defines the effects of ARID1A deficiency on outcomes in patients with EGFR-mutated LUAD, thus suggesting that ARID1A deficiency could be an independent prognostic marker and that this deficiency attenuates the response to EGFR-TKIs in patients with EGFR-mutant LUAD. In addition, loss of ARID1A expression confers resistance to EGFR-TKI by activating the EGFR/AKT/mTOR signalling axis.

## Data Availability

The original contributions presented in the study are included in the article/supplementary material, further inquiries can be directed to the corresponding authors.

## Abbreviations

LUAD, lung adenocarcinoma; ARID1A, AT-interacting domain-rich protein 1A; ORR, objective response rate; PFS, progression-free survival; SWI/SNF, switch/sucrose nonfermenting; ICIs, immune checkpoint inhibitors; ECOG, Eastern Cooperative Oncology Group; GSEA, Gene Set Enrichment Analysis; MMR, mismatch repair; TIME, tumor immune microenvironment; LOF, loss-of-function; OXPHOS, oxidative phosphorylation; RTK, receptor tyrosine kinase.

## References

[B1] Bray F. Laversanne M. Sung H. Ferlay J. Siegel R. L. Soerjomataram I. (2024). Global cancer statistics 2022: GLOBOCAN estimates of incidence and mortality worldwide for 36 cancers in 185 countries. CA Cancer J. Clin. 74, 229–263. 10.3322/caac.21834 38572751

[B2] Centore R. C. Sandoval G. J. Soares L. M. M. Kadoch C. Chan H. M. (2020). Mammalian SWI/SNF chromatin remodeling complexes: emerging mechanisms and therapeutic strategies. Trends Genet. 36, 936–950. 10.1016/j.tig.2020.07.011 32873422

[B3] Chang S. C. Lai Y. C. Chang C. Y. Huang L. K. Chen S. J. Tan K. T. (2019). Concomitant genetic alterations are associated with worse clinical outcome in EGFR mutant NSCLC patients treated with tyrosine kinase inhibitors. Transl. Oncol. 12, 1425–1431. 10.1016/j.tranon.2019.07.008 31401335 PMC6700434

[B4] de Bruijn I. Kundra R. Mastrogiacomo B. Tran T. N. Sikina L. Mazor T. (2023). Analysis and visualization of longitudinal genomic and clinical data from the AACR project GENIE biopharma collaborative in cBioPortal. Cancer Res. 83, 3861–3867. 10.1158/0008-5472.Can-23-0816 37668528 PMC10690089

[B5] Dhanyamraju P. K. (2024). Drug resistance mechanisms in cancers: execution of pro-survival strategies. J. Biomed. Res. 38, 95–121. 10.7555/jbr.37.20230248 38413011 PMC11001593

[B6] Ding W. Yang P. Zhao X. Wang X. Liu H. Su Q. (2024). Unraveling EGFR-TKI resistance in lung cancer with high PD-L1 or TMB in EGFR-sensitive mutations. Respir. Res. 25, 40. 10.1186/s12931-023-02656-3 38238740 PMC10797755

[B7] Duska L. R. Zamarin D. Hamilton E. Oza A. Fleming G. Spira A. (2023). Phase IIa study of PLX2853 in gynecologic cancers with known ARID1A mutation and phase ib/IIa study of plx2853/carboplatin in platinum-resistant epithelial ovarian cancer. JCO Precis. Oncol. 7, e2300235. 10.1200/po.23.00235 37797273

[B8] Fukuoka M. Wu Y. L. Thongprasert S. Sunpaweravong P. Leong S. S. Sriuranpong V. (2011). Biomarker analyses and final overall survival results from a phase III, randomized, open-label, first-line study of gefitinib versus carboplatin/paclitaxel in clinically selected patients with advanced non-small-cell lung cancer in Asia (IPASS). J. Clin. Oncol. 29, 2866–2874. 10.1200/jco.2010.33.4235 21670455

[B9] Grosse A. Grosse C. Rechsteiner M. (2019). Analysis of the frequency of oncogenic driver mutations and correlation with clinicopathological characteristics in patients with lung adenocarcinoma from Northeastern Switzerland. Diagn Pathol. 14, 18. 10.1186/s13000-019-0789-1 30744664 PMC6371584

[B10] Guo Y. Song J. Wang Y. Huang L. Sun L. Zhao J. (2020). Concurrent genetic alterations and other biomarkers predict treatment efficacy of EGFR-TKIs in EGFR-mutant non-small cell lung cancer: a review. Front. Oncol. 10, 610923. 10.3389/fonc.2020.610923 33363040 PMC7758444

[B11] Halaburkova A. Cahais V. Novoloaca A. Araujo M. Khoueiry R. Ghantous A. (2020). Pan-cancer multi-omics analysis and orthogonal experimental assessment of epigenetic driver genes. Genome Res. 30, 1517–1532. 10.1101/gr.268292.120 32963031 PMC7605261

[B12] Hellyer J. A. White M. N. Gardner R. M. Cunanan K. Padda S. K. Das M. (2022). Impact of tumor suppressor gene Co-mutations on differential response to EGFR TKI therapy in EGFR L858R and exon 19 deletion lung cancer. Clin. Lung Cancer 23, 264–272. 10.1016/j.cllc.2021.09.004 34838441

[B13] Hou H. Qin K. Liang Y. Zhang C. Liu D. Jiang H. (2019). Concurrent TP53 mutations predict poor outcomes of EGFR-TKI treatments in Chinese patients with advanced NSCLC. Cancer Manag. Res. 11, 5665–5675. 10.2147/cmar.S201513 31417310 PMC6594053

[B14] Hsu K. H. Huang Y. H. Tseng J. S. Chen K. C. Ku W. H. Su K. Y. (2019). High PD-L1 expression correlates with primary resistance to EGFR-TKIs in treatment naïve advanced EGFR-mutant lung adenocarcinoma patients. Lung Cancer 127, 37–43. 10.1016/j.lungcan.2018.11.021 30642549

[B15] Huang R. Wu D. Zhang K. Hu G. Liu Y. Jiang Y. (2024). ARID1A loss induces P4HB to activate fibroblasts to support lung cancer cell growth, invasion, and chemoresistance. Cancer Sci. 115, 439–451. 10.1111/cas.16052 38100120 PMC10859615

[B16] Hung Y. P. Redig A. Hornick J. L. Sholl L. M. (2020). ARID1A mutations and expression loss in non-small cell lung carcinomas: clinicopathologic and molecular analysis. Mod. Pathol. 33, 2256–2268. 10.1038/s41379-020-0592-2 32572156

[B17] Jin F. Yang Z. Shao J. Tao J. Reißfelder C. Loges S. (2023). ARID1A mutations in lung cancer: biology, prognostic role, and therapeutic implications. Trends Mol. Med. 29, 646–658. 10.1016/j.molmed.2023.04.005 37179132

[B18] Keller P. J. Adams E. J. Wu R. Côté A. Arora S. Cantone N. (2024). Comprehensive target engagement by the EZH2 inhibitor tulmimetostat allows for targeting of ARID1A mutant cancers. Cancer Res. 84, 2501–2517. 10.1158/0008-5472.Can-24-0398 38833522 PMC11292196

[B19] Kim E. S. Melosky B. Park K. Yamamoto N. Yang J. C. (2021). EGFR tyrosine kinase inhibitors for EGFR mutation-positive non-small-cell lung cancer: outcomes in Asian populations. Future Oncol. 17, 2395–2408. 10.2217/fon-2021-0195 33855865

[B20] Kim Y. Lee B. Shim J. H. Lee S. H. Park W. Y. Choi Y. L. (2019). Concurrent genetic alterations predict the progression to target therapy in EGFR-mutated advanced NSCLC. J. Thorac. Oncol. 14, 193–202. 10.1016/j.jtho.2018.10.150 30391576

[B21] Levantini E. Maroni G. Del Re M. Tenen D. G. (2022). EGFR signaling pathway as therapeutic target in human cancers. Semin. Cancer Biol. 85, 253–275. 10.1016/j.semcancer.2022.04.002 35427766

[B22] Li W. Chen Q. Gao W. Zeng H. (2022). ARID1A promotes chemosensitivity to gemcitabine in pancreatic cancer through epigenetic silencing of RRM2. Pharmazie 77, 224–229. 10.1691/ph.2022.1881 36199183

[B23] Lin Z. Li J. Zhang J. Feng W. Lu J. Ma X. (2023). Metabolic reprogramming driven by IGF2BP3 promotes acquired resistance to EGFR inhibitors in non-small cell lung cancer. Cancer Res. 83, 2187–2207. 10.1158/0008-5472.Can-22-3059 37061993

[B24] Liu Q. Yu S. Zhao W. Qin S. Chu Q. (2018). EGFR-TKIs resistance via EGFR-independent signaling pathways. Mol. Cancer 17, 53. 10.1186/s12943-018-0793-1 29455669 PMC5817859

[B25] Malone H. A. Roberts C. W. M. (2024). Chromatin remodellers as therapeutic targets. Nat. Rev. Drug Discov. 23, 661–681. 10.1038/s41573-024-00978-5 39014081 PMC11534152

[B26] Mullen J. Kato S. Sicklick J. K. Kurzrock R. (2021). Targeting ARID1A mutations in cancer. Cancer Treat. Rev. 100, 102287. 10.1016/j.ctrv.2021.102287 34619527

[B27] Offin M. Rizvi H. Tenet M. Ni A. Sanchez-Vega F. Li B. T. (2019). Tumor mutation burden and efficacy of EGFR-tyrosine kinase inhibitors in patients with EGFR-mutant lung cancers. Clin. Cancer Res. 25, 1063–1069. 10.1158/1078-0432.Ccr-18-1102 30045933 PMC6347551

[B28] Ramalingam S. S. Vansteenkiste J. Planchard D. Cho B. C. Gray J. E. Ohe Y. (2020). Overall survival with osimertinib in untreated, EGFR-mutated advanced NSCLC. N. Engl. J. Med. 382, 41–50. 10.1056/NEJMoa1913662 31751012

[B29] Rosell R. Carcereny E. Gervais R. Vergnenegre A. Massuti B. Felip E. (2012). Erlotinib versus standard chemotherapy as first-line treatment for European patients with advanced EGFR mutation-positive non-small-cell lung cancer (EURTAC): a multicentre, open-label, randomised phase 3 trial. Lancet Oncol. 13, 239–246. 10.1016/s1470-2045(11)70393-x 22285168

[B30] Sequist L. V. Yang J. C. Yamamoto N. O'Byrne K. Hirsh V. Mok T. (2013). Phase III study of afatinib or cisplatin plus pemetrexed in patients with metastatic lung adenocarcinoma with EGFR mutations. J. Clin. Oncol. 31, 3327–3334. 10.1200/jco.2012.44.2806 23816960

[B31] Skoulidis F. Heymach J. V. (2019). Co-occurring genomic alterations in non-small-cell lung cancer biology and therapy. Nat. Rev. Cancer 19, 495–509. 10.1038/s41568-019-0179-8 31406302 PMC7043073

[B32] Soria J. C. Ohe Y. Vansteenkiste J. Reungwetwattana T. Chewaskulyong B. Lee K. H. (2018). Osimertinib in untreated EGFR-mutated advanced non-small-cell lung cancer. N. Engl. J. Med. 378, 113–125. 10.1056/NEJMoa1713137 29151359

[B33] Succony L. Rassl D. M. Barker A. P. McCaughan F. M. Rintoul R. C. (2021). Adenocarcinoma spectrum lesions of the lung: detection, pathology and treatment strategies. Cancer Treat. Rev. 99, 102237. 10.1016/j.ctrv.2021.102237 34182217

[B34] Sun D. Feng F. Teng F. Xie T. Wang J. Xing P. (2023). Multiomics analysis revealed the mechanisms related to the enhancement of proliferation, metastasis and EGFR-TKI resistance in EGFR-mutant LUAD with ARID1A deficiency. Cell Commun. Signal 21, 48. 10.1186/s12964-023-01065-9 36869329 PMC9985251

[B35] Sun D. Zhu Y. Zhao H. Bian T. Li T. Liu K. (2021). Loss of ARID1A expression promotes lung adenocarcinoma metastasis and predicts a poor prognosis. Cell Oncol. (Dordr) 44, 1019–1034. 10.1007/s13402-021-00616-x 34109546 PMC12980766

[B36] Sun R. Hou Z. Zhang Y. Jiang B. (2022). Drug resistance mechanisms and progress in the treatment of EGFR-mutated lung adenocarcinoma. Oncol. Lett. 24, 408. 10.3892/ol.2022.13528 36245822 PMC9555020

[B37] Vasaikar S. V. Straub P. Wang J. Zhang B. (2018). LinkedOmics: analyzing multi-omics data within and across 32 cancer types. Nucleic Acids Res. 46, D956-D963–d963. 10.1093/nar/gkx1090 29136207 PMC5753188

[B38] Vokes N. I. Chambers E. Nguyen T. Coolidge A. Lydon C. A. Le X. (2022). Concurrent TP53 mutations facilitate resistance evolution in EGFR-mutant lung adenocarcinoma. J. Thorac. Oncol. 17, 779–792. 10.1016/j.jtho.2022.02.011 35331964 PMC10478031

[B39] Wang D. Wang J. Zhou D. Wu Z. Liu W. Chen Y. (2023). SWI/SNF complex genomic alterations as a predictive biomarker for response to immune checkpoint inhibitors in multiple cancers. Cancer Immunol. Res. 11, 646–656. 10.1158/2326-6066.Cir-22-0813 36848524 PMC10155041

[B40] Wang L. Deng C. H. Luo Q. Su X. B. Shang X. Y. Song S. J. (2022). Inhibition of Arid1a increases stem/progenitor cell-like properties of liver cancer. Cancer Lett. 546, 215869. 10.1016/j.canlet.2022.215869 35964817

[B41] Wang L. Qu J. Zhou N. Hou H. Jiang M. Zhang X. (2020). Effect and biomarker of immune checkpoint blockade therapy for ARID1A deficiency cancers. Biomed. Pharmacother. 130, 110626. 10.1016/j.biopha.2020.110626 32791396

[B42] Wu D. N. Zhang K. L. Chen R. H. Ye W. S. Zheng C. Zheng Y. L. (2024). VASN promotes the aggressive phenotype in ARID1A-deficient lung adenocarcinoma. BMC Cancer 24, 1327. 10.1186/s12885-024-13083-y 39472811 PMC11520519

[B43] Xue Y. Morris J. L. Yang K. Fu Z. Zhu X. Johnson F. (2021). SMARCA4/2 loss inhibits chemotherapy-induced apoptosis by restricting IP3R3-mediated Ca(2+) flux to mitochondria. Nat. Commun. 12, 5404. 10.1038/s41467-021-25260-9 34518526 PMC8438089

[B44] Yoodee S. Peerapen P. Plumworasawat S. Thongboonkerd V. (2021). ARID1A knockdown in human endothelial cells directly induces angiogenesis by regulating angiopoietin-2 secretion and endothelial cell activity. Int. J. Biol. Macromol. 180, 1–13. 10.1016/j.ijbiomac.2021.02.218 33675830

[B45] Zhang X. Zhang Y. Zhao J. Wu Y. Zhang N. Shen W. (2023). ARID1A mutations in cancer development: mechanism and therapy. Carcinogenesis 44, 197–208. 10.1093/carcin/bgad011 36882165

